# Diagnostic Accuracy of Flow Cytometric DNA Index in Saudi Children with B Cell Acute Lymphoblastic Leukemia

**DOI:** 10.3390/children10081299

**Published:** 2023-07-28

**Authors:** Dalal Alatawi, Ismail Bakhsh, Soha Tashkandi, Abdullah Alqarni, Abjal Pasha Shaik, Manal Abudawood

**Affiliations:** 1 Department of Laboratory and Blood Bank (Hematology), King Fahad Specialist Hospital, Tabuk 47717, Saudi Arabia; 2 Department of Clinical Laboratory Science, College of Applied Medical Sciences, King Saud University, Riyadh 11433, Saudi Arabia; ashaik@ksu.edu.sa; 3 Green Crescent Hospital, Riyadh 12711, Saudi Arabia; 4 Department of Cytogenetic Laboratory, King Fahad Medical City, Riyadh 11525, Saudi Arabia; 5 Department of Flow Cytometry Laboratory, King Fahad Medical City, Riyadh 11525, Saudi Arabia; 6 Chair of Medical and Molecular Genetics Research, Department of Clinical Laboratory Sciences, Collage of Applied Medical Sciences, King Saud University, Riyadh 11433, Saudi Arabia

**Keywords:** DNA Index, flow cytometry, ploidy, B cell ALL, childhood leukemia, hyperdiploid, aneuploidy

## Abstract

B cell Acute Lymphoblastic Leukemia (B-ALL) is one of the most common types of cancer diagnosed in children in Saudi Arabia. Cytogenetic investigations, including karyotyping and FISH, are used to determine the incidence and prognostic significance of chromosomal abnormalities in B-ALL. However, in ALL, accurate identification of the morphology of chromosomes is sometimes not achieved. Flow cytometric DI may have the advantage of being technically fast, using either fresh or frozen samples to correctly stratify the patient into the appropriate risk group for treatment. In this study, we evaluated the reliability and validity of using fixed samples instead of fresh samples to determine aneuploidy in cancer cells using a DNA index. The results of the DNA index obtained by flow cytometry were compared with those of conventional cytogenetic analysis to validate the accuracy. Fixed samples (n = 72) from children diagnosed with B-ALL at King Fahad Medical City in Riyadh between 2017 and 2019 were investigated. The results showed strong and statistically significant positive correlation between DNAI-FCM and conventional cytogenetic analysis (*p =* 0.000 < 0.01). The DNA index value by flow cytometry was proportional to the cytogenetic study in 94.36% (67) of the cases, while discrepancy was observed in 5.6% (four) cases. Our findings highlight the ability of the DNA index method to provide complementary information for the accurate diagnosis of aneuploidy in patients with B-ALL.

## 1. Introduction

Determining the DNA content of blast cells is an important diagnostic and prognostic factor in childhood B-ALL. Two conventional methods are commonly used to investigate the ploidy of leukemia blasts. The first method involves karyotyping cultured bone marrow cells using light microscopy to count Giemsa-banded metaphase chromosomes. This method is now combined with analyses using fluorescence in situ hybridization (FISH) to enhance its sensitivity [1]. However, this method is relatively insensitive due to the low number of metaphases studied. At least 2 cells showing the same mandatory anomalies are required from up to 20 metaphases studied to call a relative numerical change, and 3 cells are necessary in case of total chromosome loss. The second approach involves measuring the DNA content by either quantitative microscopic image analysis or flow cytometry [2,3]. The DNA Index (DI) by flow cytometry method is considered more sensitive than karyotyping, because it can analyze large numbers of cells and detect aneuploid clones that represent only 2–3% of the cell population studied [4,5].

In childhood B-ALL, determining the presence of aneuploid cells and estimating the growth fraction of the population is crucial, and the DI is an important diagnostic tool for this purpose [6]. Studies have found a strong correlation between the number of chromosomes detected using karyotyping and the DNA contents detected by flow cytometry in fresh and frozen samples [4,5]. While DI was able to detect aneuploid leukemic clones in 36.8% (7/19) ALL children at day 15 and 29, karyotyping in all of the follow-up samples were diploid [4]. Karyotyping diagnosed biclonality in two patients that was not detected at diagnosis, while DI detected abnormal cloning during the first month of induction therapy in over 20% of aneuploid patients, which had diploid karyotype [6]. Furthermore, the cytogenetic approach only discovered the hyperploid clone in three patients presenting the near-triploid/hyperploid entity, while DI identified a minor population of hypodiploid cells, besides the major hyperdiploid clone, thereby validating the diagnosis of severe hypodiploidy [6]. However, the sensitivity of these techniques to detect small DNA content abnormalities depends on the quality of the sample, the staining technique, and the instrument used [4].

Flow cytometric DI has been used as a prognostic factor in childhood ALL. Studies have suggested that measurement using DI is a reliable method to assess prognosis, with statistically significant differences in survival rates observed using the DI approach as opposed to modal numbers obtained through karyotyping [7,8]. Approximately 25–30% of children with ALL who have a DI ≥ 1.16 (corresponding to a hyperdiploid modal number of >50 chromosomes) have more favorable prognoses and higher cure rates than other major prognostic subgroups [5]. Conversely, hypodiploid clones (<44 chromosome) are associated with poor diagnoses [9]. In a diagnostic study of 40 children with B-ALL, DNA ploidy was assessed using flow cytometry [10]. Most cases were found to be aneuploid (65% [26/40]), while the remaining were diploid (35% [14/40]), with a DI between 0.90–1.05.

Furthermore, 50% of cases had a DI between 1.10–1.6 (hyperdiploid B), and 15% had a DI > 1.6 (triploid and tetraploid range). None of the cases had a DI < 0.90 (hypodiploidy), or a DI between 1.06–1.09 (hyperdiploid A). A correlation between the conventional cytogenetic result and DI by flow cytometry in the paediatric population with B-ALL was found [3]. Karyotyping classified cases as: Diploid (10.53% [6/57]), pseudodiploid (36.84% [21/57]), hyperdiploid (22.81% [13/57]), and high hyperdiploid (36.84% [17/57]). While DI provided a significant correlation with the conventional cytogenetic results in most cases, there were discrepancies in 12 out of 69 cases [3]. These findings suggest that DI is a reliable and sensitive method for determining cell ploidy. Another study that analyzed 588 children diagnosed with ALL over 10 years indicated that the 3 techniques (karyotype, FISH, and DI) in combination helps to determine the correct genetic subgrouping, especially in the case of lack of successful karyotyping, or if the cells have more than one cell line [11].

We assessed the reliability and validity of using fixed samples instead of fresh samples in determining aneuploidy in cancer cells by using the DNA index via flow cytometry method. The accuracy of the DNA index was verified by correlating the values obtained with chromosome numbers from cytogenetic testing.

**SIGNIFICANCE OF THE STUDY**.

The genetic classification of B-ALL is essential for risk group stratification and in treatment evaluation. The discovery of genetic variables can be used to develop diagnostic and prevention tools to reduce genetic diseases and promote public health. Furthermore, such information will enable the improvement of the diagnostic tests for B-ALL in children. This is in line with the Saudi government’s program, Saudi Vision 2030, which aims, in part, to achieve whole genome sequencing for the Saudi population, in addition to building a genetic database for Saudi society, by identifying the genes that can cause genetic or inherited diseases. The current study’s findings will help facilitate an understanding of the common cytogenetic abnormalities behind B-ALL, specifically in Saudi children.

## 2. Materials and Methods

We collected 72 samples from Saudi children newly diagnosed with B cell acute lymphoblastic leukemia at King Fahad Medical City in Riyadh over a period of three years (2017–2019) Cytogenetic results were obtained using karyotyping and FISH. Samples collected from cytogenetic laboratory were fixed with formalin and tested for DNA index using flow cytometry (July 2020), as per manufacturer’s instructions. After checking the instrument linearity using the CEN (cat# 349523) and CTN (cat# 349523) vials provided by the company, prepared patient samples (fixed cells, (the initial WBC count is ~10 × 10^9^/L for optimum stain saturation and to avoid false hypodiploidy results) were stained with propidium iodide (PI) and samples were loaded into the flow cytometer. A normal blood control was run each time as an internal QC.

The instrument used for running samples and analyses was the BD FACS Canto^TM^ II machine, and the calibration of the instrument was performed using FACSDiva Software. Analysis of the DNA histogram was performed by the MOD fit LTV4.0.5 software. Descriptive and inferential analyses were performed. The correlation between the DNA index value and the number of chromosomes obtained by conventional cytogenetics was measured via Pearson’s correlation using the IBM SPSS Statistics ver. 26, 2019 software.

## 3. Results

The patient characteristics and cytogenetic results are summarised in Table 1. Of the 72 patients, 43 (59.7%) were male and 29 (40.3%) were female. Most of the samples were from bone marrow (93.1%), and the rest were from leukemic blood (6.9%). A total of 144 patients underwent chromosome analysis (65.3%) and FISH (91.7%).

The results in Table 1 show that 29.2% failed the chromosome analysis test while none failed the FISH test, and four cases had only the FISH results, while six had only the karyotype result. Of these cases, 38.9% were found to be abnormal under chromosome analysis, 64.3% showed numerical abnormalities, while 35.7% showed structural abnormalities. Karyotyping (Table 2) was successful in the metaphases of 47 cases (diploid [n = 19 patients], pseudodiploid [n = 10], hypodiploid [n = 1], high hyperdiploid [n = 15], and low hyper-diploid [n = 2]). However, FISH detected abnormalities in 84.9% of the samples, with numerical and structural abnormalities accounting for 41.78% and 39.15%, respectively (Table 3). The most common anomalies detected were hyperdiploid (42.4%), BCR/ABL1 (4.5%), ETV6/RUNX1 (21.2%), and MLL rearrangement (3.03%).

All patients had one large cell clone (the “dominant” clone, corresponding to 50% to 100% of the analysed cell population). Of the 72 patients, only 1 (1.4%) was excluded owing to the high aggregate percentage of 96% in the DNA fluorescence histogram (Table 4). Of the remaining 71 cases, 42 (58.3%) had a dominant diploid clone corresponding to 100% of the analysed cell population and 27 patients (37.5%) had a dominant high hyperdiploid clone with DI between 1.10 and 1.6. Two patients had a tetraploid clone: one patient (DI of 1.99, corresponding to 8.94% of the analysed cell population at G2-M channel) and the second patient (DI of 1.94, corresponding to 4.47% of the analysed cell population at G2-M channel) did not represent the aneuploid clone. Among the 27 patients with a dominant high hyperdiploid clone, 2 patients had an additional tetraploid leukemic subclone, with DIs of 2.28 and 2.58 (tetraploid DNA index between 1.6 and 2.1), representing 6.52% and 19.14% of the analysed cell population, respectively. This subclone was considered as the cell in G2-M phase channel, which meant that it was not a new aneuploid clone. All 27 patients with a dominant high hyperdiploid or tetraploid clone had a subset diploid clone constituting between 13% and 90% of the total amount of analysed cells. 

Out of 46 karyotype results, 43 (93.47%) matched with the results of the DI, while 3 (6.5%) did not (1 sample showed 45 and 47 chromosomes [hypodiploid and hyperdiploid A], while two showed 47 chromosomes). However, out of the 65 FISH results, 61 (93.84%) matched with the DI while 4 (6.1%) did not.

Using automatic analysis alone, there were matches in results between the DI and cytogenetic tests in 52 cases (73.24%), while 19 cases (26.76%) did not match. Of these no matches, 13 cases were high hyperdiploid B (>51 chromosomes), 1 case failed by karyotyping and FISH was TCF3/PBX1 but was triploid by DI at the G2-M channel, and five cases were hyperdiploid A (47–51 chromosome). To resolve this discrepancy in results, we used manual analysis instead of automatic analysis only for the DI histogram analysis of 19 cases, by adjusting the software to analyse 2 cell cycles for more accurate detection of any aneuploidy population of the cells. Manual analysis for the 2-cell cycle by the MOD fit software of the 19 discrepant cases showed 16 cases had a hyper-diploid clone corresponding to 1.39% to 99.25% of the analysed cell population (DI 1.10 to 1.16). Two cases had a tetra-ploidy clone corresponding to 4.47% and 2.85% at G2-M channel (considered a not aneuploid clone because at this phase of the cell cycle, the cells harbor double the number of chromosomes), and one case had a hyperdiploid A clone corresponding to 75.74% of the analyzed cells (DI 1.09). The DI values in the aneuploid and diploid groups were as follows: 42 (59.1%) had DI 1–1.05, 26 (36.6%) had DI 1.10–1.6, and 3 (4.2%) had DI > 1.6 (Table 5).

**Figure 1:**
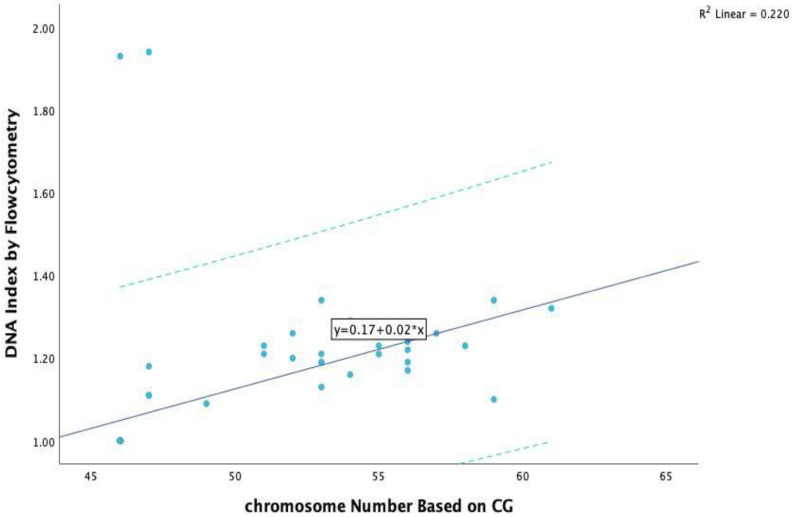
The linear relation between chromosome number obtained by cytogenetics test and DNA index value by automatic and manual analysis by MOD fit software.

In summary, by using manual analysis, 15 out of the 19 cases were matched with cytogenetics and the discrepancies were resolved. Of the 71 cases, 67 (94.37%) cases matched with cytogenetics while 4 (5.6%) did not. A correlation analysis was to determine an association between the DI value (by automatic and manual analysis) and the chromosome number via cytogenetics tests (karyotype or FISH), and showed that the DI value was positive, strong, statistically significant, and correlated with the number of chromosomes obtained based on the cytogenetics tests (r = 0.469, *p* value = 0.000 < 0.01) (Figure 1).

## 4. Discussion

Aneuploidy, a change in the ploidy or number of chromosomes within the cell, is one of the cytogenetic abnormalities detected in children with B-ALL. Aneuploidy can be detected by many techniques such as karyotyping, interphase cytogenetics (FISH), and DI (flow cytometry). Flow cytometry with image analyses can detect aneuploidy by measuring the relative DNA content of the cell with respect to the reference diploid cells [12]. While karyotyping is the standard technique used to evaluate ploidy, it has some limitations, such as requiring living cells to spontaneously enter mitosis, and it can take up to 28 days to provide a diagnosis. FISH, on the other hand, uses specific probes to target chromosomes, but it can be expensive and cannot evaluate all chromosome statuses, except when multiple probes are used. In contrast, DNA analysis by flow cytometry is easy to perform on living or fixed cells, independent of cell proliferation, and can be completred within two hours. DI analysis by flow cytometry can detect small aneuploidy clones that make up a low percentage of the total cell population and can analyze thousands of cells per minute.

The aim of this study was to evaluate the reliability of three techniques used to detect aneuploidy by studying the association between the DI value and chromosome numbers obtained by cytogenetics techniques. Studies from Saudi Arabia that assessed the impact of these techniques on the diagnostic accuracy are limited. The study used formalin-fixed cells collected from culturing bone marrow or leukemic blood samples. DI analysis was conducted on 72 children diagnosed with B-ALL.

Flow cytometric analysis for DNA ploidy was performed on all 72 children. Of the 72 cases, 1 case was excluded due to a high percentage of aggregates (96%). Karyotyping was successful in 47 cases (diploid status [29 patients], hypodiploid [1 patient], high hyperdiploid [15 patients], and low hyperdiploid [2 patients]). It is worth noting that the prevalence of aneuploidy in this study is different from other studies such as Almozain et al. [3] and Forestier et al. [4]). The common cytogenetic anomalies detected in our patients were hyperdiploid, BCR/ABL1, ETV6/RUNX1, and MLL rearrangement.

In this study, the DI range was 1 to 2.58, with a median of 1.16. This finding is different from those reported in previous studies, such as Almozain et al. [3], which found a DI value range of 0.8 to 1.85, with a median of 1.09, and Kumar et al. [10], which found a DI range of 0.91 to 2.04, with a median of 1.14. Of the 71 cases in our study, 29 (40.9%) had an aneuploid stem line and 42 (59.1%) had a diploid stem line. These findings differ from those reported by Kumar et al. [10], where 65% of the evaluated 40 children showed aneuploidy and 35% showed diploidy. Similarly, in a study by Almozain et al. [3], 46.4% of cases showed aneuploidy, while 47.37% showed diploidy and pseudodiploidy, which is greater than the percentage of aneuploidy in our study. Forestier et al. [4] reported that 42% of the children had aneuploidy abnormalities with acute leukaemia. 

In our study, we took a DI value of ≥1.10 as a cut-off for high hyperdiploidy, based on the classification system used by Basu et al., 2009 [13]. Our study found that 36.6% of cases had a DI value between 1.10–1.16 and a distinct aneuploidy peak, which is comparable to the findings reported by Kumar et al. [10], who reported a DI of 1.10–1.16 in 50% of aneuploidy cases. Moreover, in our study, the prevalence of high hyperdiploidy (DI 1.10–1.6) was different from other studies, which showed a range greater than 20% [3,10]. One case in our study had a DI of 1.09, which is hyperdiploid A or less hyperdiploidy, and none of the cases in our study had a DI < 0.90 (hypodiploidy).

According to Almozain et al. [3], there is a significant correlation between DI flow cytometry and conventional cytogenetics (*p* = 0.001), and Forestier et al. [4] also reported a significant correlation between modal chromosome numbers and DI flow cytometry (*p* = 0.009). In our study, we found a significant correlation between DNAI-FCM and conventional cytogenetics (*p* = 0.000), which is consistent with the findings of Rachieru-Sourisseau et al., [5] who demonstrated a significant correlation between karyotyping and DNA content detected by flow cytometry in fresh and frozen samples.

The DI value by flow cytometry was proportional to the cytogenetic study in 94.36% (67 cases) of the cases, with only 5.6% (four cases) showing discrepancies, a value that is lower than that reported by Almozain et al. [3], where discrepancy was observed in 17.4% (12) of the cases, and Forestier et al. [4], where a discrepancy was reported in 18.29% (15) of the cases. The DI by flow cytometry can detect aneuploidy in haematological and non-haematological tumours. The discrepancies between DI value by flow cytometry and conventional cytogenetics in solid tumours were much higher, reaching 70% [14]. Most of the observed discrepancies happened with hypodiploidy cases [14,15].

Chromosome count is considered one of the important prognostic markers in B-ALL. Although high hyperdiploids (>50 chromosomes) have been related to better outcomes, [16], hypodiploids (<46 chromosome) are related to worse prognosis and treatment failure. It is a rare classification seen in 5–8% of Childhood B-ALL, and sometimes may be masked by re-duplication. Hence, it is classified based on the type of chromosomes within the metaphases rather than the count alone [9]. Hypodiploidy was detected by cytogenetics in two cases only on our study, fixed sample was available for DI evaluation from one of them only, however as mentioned earlier the results showed discrepancy. Hypodiploidy is challenging in nature; from one side, it is a rare finding, and from the other side, it may be masked by endoreplication, i.e., “masked hypodiploidy”. In such cases, karyotype analysis on cultured metaphases where chromosomes can be visualized under the microscope to be counted and identified remains the currently most reliable method [17]. Some studies combined cytogenetics and DNA index in the assessment of chromosome ploidy and were able to detect hypodipolid patterns with a DNA Index of 0.55 [17,18] with more reliability in hyperdiploid subtype pick up. In our study, four cases were hyperdiploid A (47–50 chromosome) with trisomy 21 or tetrasomy. Almozain et al. [3] reported 12 cases showing a discrepancy in the chromosome number, ranging from 47–50; 4 cases had insufficient cells, while 5 had tetrasomy or trisomy 21. This discrepancy can be attributed to the small amount of genetic material represented by chromosome 21, as previously demonstrated by Morton [19]. Of the 82 cases, 15 showed discrepancy in the study of Forestier et al. [4], 9 cases with modal chromosome numbers between 47–51 had a DI value = 1, which is similar to our study, 1 case was pseudodiploidy and had a DI > 1, and 5 cases were diploidy with the karyotype showing an aneuploid clone with DI > 1.

The discrepancies could be due to the identification of hypodiploidy, which is known to cause a discrepancy between DI by flow cytometry and cytogenetics techniques [3,13]. In addition, the identification of three hyperdiploid cases with modal chromosome numbers (47–51) and trisomy or tetrasomy 21, and the diploid case, which revealed a tetraploid clone by DI at the G2-M channel, representing the same cell population with a diploid clone at the G0–G1 channel.

DI by flow cytometry is a sensitive and reliable technique for the identification of hyperdiploid leukaemia (>51 chromosomes) [3,4,5,10] and can also detect biclonality in leukaemia patients with aneuploidy [4]. The differences in DI values observed in various studies could be attributed to variations in the patient population, sample preparation, and analysis techniques. Additionally, different reference diploid cell populations used in different studies could contribute to the observed variability in DI values. It must be noted that the DI method alone may not be sufficient for the detection of masked hypodiploidy where the hypodiploid cells are present at low levels and are difficult to detect by flow cytometry alone. Additional methods such as FISH or molecular genetic techniques may be required for a more accurate and sensitive analysis. The current study was also not designed or adequately powered to analyse the correlation between DI versus other clinicopathological conditions, including survival or response. However, the literature indicates that chromosome count is a prognostic marker in B-ALL, with high hyperdiploids (>50 chromosomes) related to better outcomes, and hypodiploids (<46 chromosome) to worse prognosis and treatment failure. Despite these factors, DI by flow cytometry remains a valuable technique for detecting aneuploidy in children with B-ALL.

## 5. Conclusions

A linear correlation was observed between DI and cytogenetics studies, indicating its reliability to detect aneuploidy and biclonality. In addition, it is able to illustrate the lack of a proliferation clone, which cannot be detected by conventional cytogenetics. The use of both automatic and manual analysis by MOD fit can enhance the result of the DNA index. However, further studies need to be conducted to validate the reliability of this technique to detect hypodiploid clones. The DI test can provide complementary information in aneuploid ALL, either by confirming cellular cytogenetic data, or by detecting additional clones that have not been identified when using only cytogenetic methods. Therefore, a combination of cytogenetics and DI analysis is needed for a comprehensive genetic evaluation of B-ALL patients. Overall, the results of this study suggest that DI analysis by flow cytometry is a valuable tool for detecting aneuploidy in B-ALL and can provide additional information to aid in the diagnosis and prognosis of this disease.

## Figures and Tables

**Table 1:** Patient characteristics and cytogenetic results.

Parameter		
Total sample size		72 (100%)
Gender	Male	43 (59.7%)
	Female	29 (40.3%)
Age range Median +/− SD		0–14 years 5.40 +/− 3.789
Type of sample	Bone marrow	67 (93.1%)
	Leukemic blood	5 (6.9%)
Karyotype	Total	47/72 (65.3%)
	Normal	19 (26.4%)
	Abnormal	28 (38.9%)
	Failed	21 (29.2%)
	No result (ONLY FISH)	4 (5.6%)
FISH	Total	66/72 (91.7%)
	Normal	10 (13.8%)
	Abnormal	56 (77.8%)
	Failed	-
	No result (ONLY karyotype)	6 (8.3%)

**Table 2:** Cytogenetic result of karyotype.

Karyotype	n (%) 47
Normal	19 (40.4%)
Pseudodiploid (Chromosomal rearrangement: BCR/ABL, 4 case; Deletion 9, 4 cases; TCF3/PBX1, 1 case; KMT2A/MLLT1, 1 case)	10 (21.27%)
Hyperdiploid A (47–50)	2 (4.2%)
Hyperdiploid B (51–68)	15 (31.91%)
Hypodiploid <46	1 (2.1%)

**Table 3:** Cytogenetic result of FISH.

FISH	n (%) (Total = 66)
Normal	10 (15.2%)
Hyperdiploid A (47–50) Pseudo hyperdiploid A	3 (4.5%) 2 (3.03%)
Hyperdiploid B (51–68) Pseudo hyperdiploid A	22 (33.4%) 1 (1.5%)
BCR/ABL	3 (4.5%)
MLL Rearrangement	2 (3.03%)
ETV6/RINX1	14 (21.2%)
TCF3/PBX1	2 (3.03%)
CDNK2A deletion	6 (9.1%)
RUNX1 amp	1 (1.5%)

**Table 4:** DNA Index result by flow cytometry automatic and manual analysis.

Type of Analysis	DNA Index Result	Category of Case Based on DI	Category of Case Based on CG	Status
Automatic	1.17/2.28	Hyperdiploid B/Tetraploid	Hyperdiploid B	Match
	1.19	Hyperdiploid B	Hyperdiploid B	
	1.19	Hyperdiploid B	Hyperdiploid B	
	1.21	Hyperdiploid B	Hyperdiploid B	
	1.22	Hyperdiploid B	Hyperdiploid B	
	1.24	Hyperdiploid B	Hyperdiploid B	
	1.26	Hyperdiploid B	Hyperdiploid B	
	1.26	Hyperdiploid B	Hyperdiploid B	
	1.32/2.58	Hyperdiploid B/Tetraploidy	Hyperdiploid B	
	1.34	Hyperdiploid B	Hyperdiploid B	
	1.99	Tetraploid	Diploid	
Manual	1.13	Hyperdiploid B	Hyperdiploid B	Match
	1.18	Hyperdiploid B	Hyperdiploid A	No Match
	1.11	Hyperdiploid B	Hyperdiploid A	
	1.11	Hyperdiploid B	Hyperdiploid B	
	1.94	Tetraploid	Hyperdiploid A	
	1.11	Hyperdiploid B	Hyperdiploid A	
	1.21	Hyperdiploid B	Hyperdiploid B	Match
	1.34	Hyperdiploid B	Hyperdiploid B	
	1.27	Hyperdiploid B	Hyperdiploid B	
	1.16	Hyperdiploid B	Hyperdiploid B	
	1.10	Hyperdiploid B	Hyperdiploid B	
	1.20	Hyperdiploid B	Hyperdiploid B	
	1.23	Hyperdiploid B	Hyperdiploid B	
	1.09	Hyperdiploid A	Hyperdiploid A	
	1.23	Hyperdiploid B	Hyperdiploid B	
	1.21	Hyperdiploid B	Hyperdiploid B	
	1.23	Hyperdiploid B	Hyperdiploid B	
	1.24	Hyperdiploid B	Hyperdiploid B	
Automatic 42 CASES	1	Diploid 42 CASES	Diploid 42 CASES	Match 42 CASES
		Total	71	
	The sample was highly aggregating 96%		1	Exclude
		Total	72	

**Table 5:** The Cytogenetic Data of Case that are NOT Matched with the Result of DNA Index Value by Flow Cytometry.

Case Number	Test	ISCN	Category of Abnormalities	Number of Chromosomes Based on Cytogenetic Test	DNA Index by MOD Fit Software
1	Karyotype	47,XXY?c[20]	Hyper-diploid A +X	47	1.18
	FISH	nuc ish(PBX1,TCF3)x2[200], (D4Z1,D10Z1)x2[200], (D6Z1x2)[200], (ABL1,BCR)x2[200], (CDNK2Ax2)[200], (MLLx2)(5′sep 3′MLLx1)[200], (ETV6,RUNX1)x2[200], (TP53x2)[200]	MLL rearrangement		
2	Karyotype	47, XX,+21[4]/46, XX[16]	Hyper-diploid A +21	47 46	1.11
	FISH	nuc ish(PBX1,TCF3)x2[200], (D4Z1,D10Z1)x2[200], (D6Z1x2)[200], (ABL1,BCR)x2[200], (CDNK2Ax2)[200], (MLLx2)[200], (ETV6x2,RUNX1x3)[170/200], (TP53x2)[200]	Hyper-diploid A	47	
3	Karyotype	No result	_	_	1.94
	FISH	nuc ish(PBX1,E2A)x2[200], (D4Z1,D10Z1)x2[200], (D6Z1x3)[168/200], (ABL1,BCR)x2[200], (CDNK2Ax2)[200], (MLLx2) (5′ MLL sep 3′MLLx1)[180/200], (ETV6,RUNX1)x2[200], (TP53x2)[200]	Hyper-diploid A MLL rearrangement	47	
4	Karyotype	45,XX,t(9;22)(q34;q11.2),add(13)(p11.2),−20[9]/ 47, XX,t(9;22)(q34;q11.2),+der(22)t(9;22)(q34;q11.2)[6]	Hypodiploid Hyper-diploid A	45 47	1.11
	FISH	nuc ish(PBX1,TCF3)x2[200], (D4Z1,D10Z1)x2[200], (D6Z1x2)[200], (ABL1x3),(BCRx3), (ABL1con BCRx2)[158/200], (D9Z1,CDNK2A)x2[200], (MLLx2)[200],(ETV6,RUNX1)x2[200], (TP53x2)[200]/ (ABL1x4),(BCRx4), (ABL1con BCRx3)[42/200]	Hyper-diploid A BCR/ABL	48	

## Data Availability

Data sharing not applicable due to privacy.
